# Endoscopic enucleation of the prostate versus transurethral resection of the prostate for benign prostatic hyperplasia: a systematic review and meta-analysis

**DOI:** 10.1038/s41391-025-00970-z

**Published:** 2025-05-10

**Authors:** Lequang T. Vo, David Armany, Venu Chalasani, Simon V. Bariol, Sriskanthan Baskaranathan, Tania Hossack, David Ende, Henry H. Woo

**Affiliations:** 1 Department of Urology, Blacktown Mount-Druitt Hospital, Sydney, NSW Australia; 2 School of Medicine, Western Sydney University, Sydney, NSW Australia; 3 School of Medicine, University of Sydney, Sydney, NSW Australia

**Keywords:** Prostatic diseases, Outcomes research

## Abstract

**Background:**

Endoscopic enucleation of the prostate (EEP) has emerged as a leading surgical treatment for benign prostatic hyperplasia (BPH), traditionally managed by transurethral resection of the prostate (TURP). EEP involves complete adenoma removal along the surgical capsule and can be performed using different energy sources, such as holmium, thulium, GreenLight and diode lasers, or bipolar electrocautery. This meta-analysis compares the efficacy and safety of EEP versus TURP.

**Methods:**

A comprehensive search of MEDLINE, EMBASE, CENTRAL, Web of Science, and Scopus (2003-present) identified randomised controlled trials (RCTs) comparing EEP with TURP in adult males (≥18 years) with BPH. Primary outcomes comprised functional measures (Qmax, PVR, IPSS, QoL, IIEF-5), while secondary outcomes included adverse events (incontinence, bleeding, infection, re-treatment rates, hospital stay duration). Two reviewers independently performed data extraction and assessed risk of bias using the Cochrane RoB2 tool.

**Results:**

Twenty-eight RCTs (*n* = 3085) met inclusion criteria: 1538 patients underwent EEP and 1547 underwent TURP. EEP was associated with significantly improved IPSS (at 12 months), Qmax (1, 6, 12, 24 months), and PVR (6, 12, 36 months) compared with TURP. Perioperative outcomes favoured EEP, including shorter catheterisation time (MD = −1.12 days), reduced hospital stay (MD = −0.92 days), and lower transfusion rates (RR = 0.22). No significant differences were observed in long-term incontinence or bladder neck contracture, though EEP yielded lower stricture rates (RR = 0.55) and reoperation rates for recurrent BPH (RR = 0.32). Heterogeneity was high in several outcomes, reflecting variability in patient characteristics, enucleation techniques, and surgeon experience.

**Conclusions:**

EEP compares favourably with TURP for BPH, providing notable benefits in bleeding control, faster recovery and durable obstruction relief. Anatomical enucleation yields functional outcomes at least equal and often superior to TURP. Energy source choice may reflect resources and surgeon preferences. Future research should distinguish enucleation completeness from energy source.

## Introduction

Endoscopic enucleation of the prostate (EEP) has become a leading option for the surgical treatment of benign prostatic hyperplasia (BPH), which was traditionally managed with transurethral resection of the prostate (TURP) [1–3]. Originally developed as an endoscopic alternative to open prostatectomy for large prostates, EEP has since demonstrated its effectiveness as a size-independent technique, offering comparable or superior outcomes to TURP in terms of both efficacy and safety [1–4].

EEP involves anatomical enucleation along the prostate’s surgical capsule, resulting in a significant reduction in residual adenoma volume. While various energy sources—such as holmium laser, thulium fibre laser, thulium:YAG laser, GreenLight laser, diode laser, and bipolar energy – are employed, the anatomical principle of enucleation remains consistent across all approaches [4].

This review equates the various energy sources used in EEP, focusing specifically on the overall efficacy and safety of the enucleation technique itself. Our meta-analysis aims to compare EEP and TURP in terms of functional outcomes and adverse events.

## Methods

### Study design

**Fig. 1:**
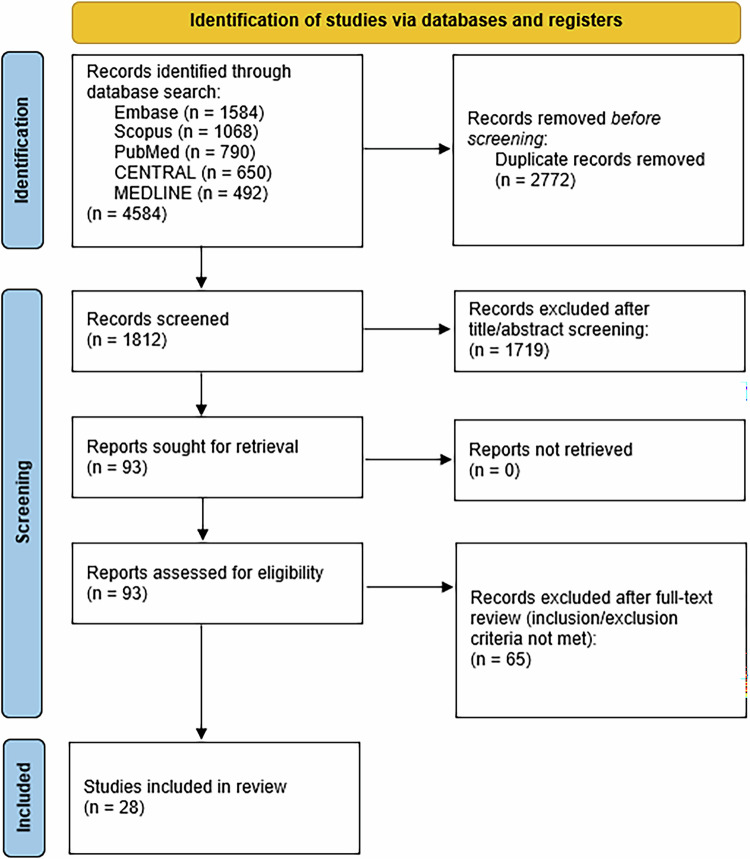
Study selection and characteristics. PRISMA flow diagram illustrating the process of record screening and study selection.

This systematic review and meta-analysis was carried out according to the Preferred Reporting Items for Systematic Reviews and Meta-Analysis (PRISMA) guidelines (Fig. 1), and the search strategy and review protocol was published in PROPSERO (Registration Number: CRD42024514177). This review focused on randomised controlled trials (RCTs) comparing EEP using various energy sources with TURP for the treatment of BPH.

### Eligibility criteria

- Population: Adult male patients (≥18 years) with BPH
- Interventions: EEP using any energy source, including but not limited to holmium laser enucleation (HoLEP), thulium:YAG laser enucleation (ThuLEP), thulium fibre laser enucleation (ThuFLEP), GreenLight vapoenucleation (GreenVEP), diode laser enucleation (DiLEP), and bipolar enucleation (BPEP)
- Comparison: TURP, performed using either monopolar or bipolar energy
- Outcomes: functional outcomes (Qmax, PVR, IPSS, QoL, IIEF-5), adverse events (urinary incontinence, bleeding, infection, re-treatment rates, length of hospital stay)
- Exclusion criteria: any resection, ablation or vapourisation technique; any non-randomised controlled trials or observational studies; any studies involving patients with prostate cancer, prior prostate interventions, concurrent bladder stones or neurogenic bladder; studies with incomplete reporting of outcomes

### Search strategy

We undertook a comprehensive literature search of electronic databases including MEDLINE, EMBASE, Cochrane Central Register of Controlled Trials (CENTRAL), Web of Science and Scopus from inception to the present date. Search terms included a combination of Medical Subject Headings (MeSH) and free-text terms related to benign prostatic hyperplasia, endoscopic enucleation of the prostate and TURP (Appendix 1). Additional sources such as ClinicalTrials.gov and hand-searching of reference lists were utilised to ensure comprehensive coverage. We excluded any articles that were not randomised controlled trials of EEP and TURP.

### Study selection

Two independent reviewers (LV and DA) screened the titles and abstracts of identified studies against the pre-defined inclusion criteria. In the case of disagreement, the article was included for a full-text review. After title and abstract review, LV and DA independently performed a full-text review and excluded articles that did not fulfil the criteria. Any disagreements at this stage were subject to discussion and when unable to reach a consensus, HHW made the final decision.

### Data extraction and analysis

Within included studies, we recorded the energy source as reported by the original authors. Where possible, we specify the form based on study details. We applied no upper or lower limits on prostate size in the search criteria to ensure broad inclusion. While baseline prostate volumes were recorded, a formal subgroup analysis based on size was not performed due insufficient standardised data.

## Results

### Study selection and characteristics

28 randomised controlled trials published between 2003 and 2024 were included in the final review. The PRISMA flow diagram is illustrated in Fig. 1. A combined total of 3085 participants were analysed, with 1538 undergoing EEP and 1547 undergoing TURP. A summary of key characteristics is shown in Supplementary Table 1 [5–32].

Participants in the EEP group had a mean age of 69.52 compared to 69.02 in the TURP group and a mean prostate volume to 73.94cc compared to 70.53cc. Follow-up duration varied across studies: 7 assessed outcomes in the short term (≤6 months), 16 in the medium-term (6–24 months) and 5 studies in the long term (>24 months).

### Risk of bias

**Fig. 2:**
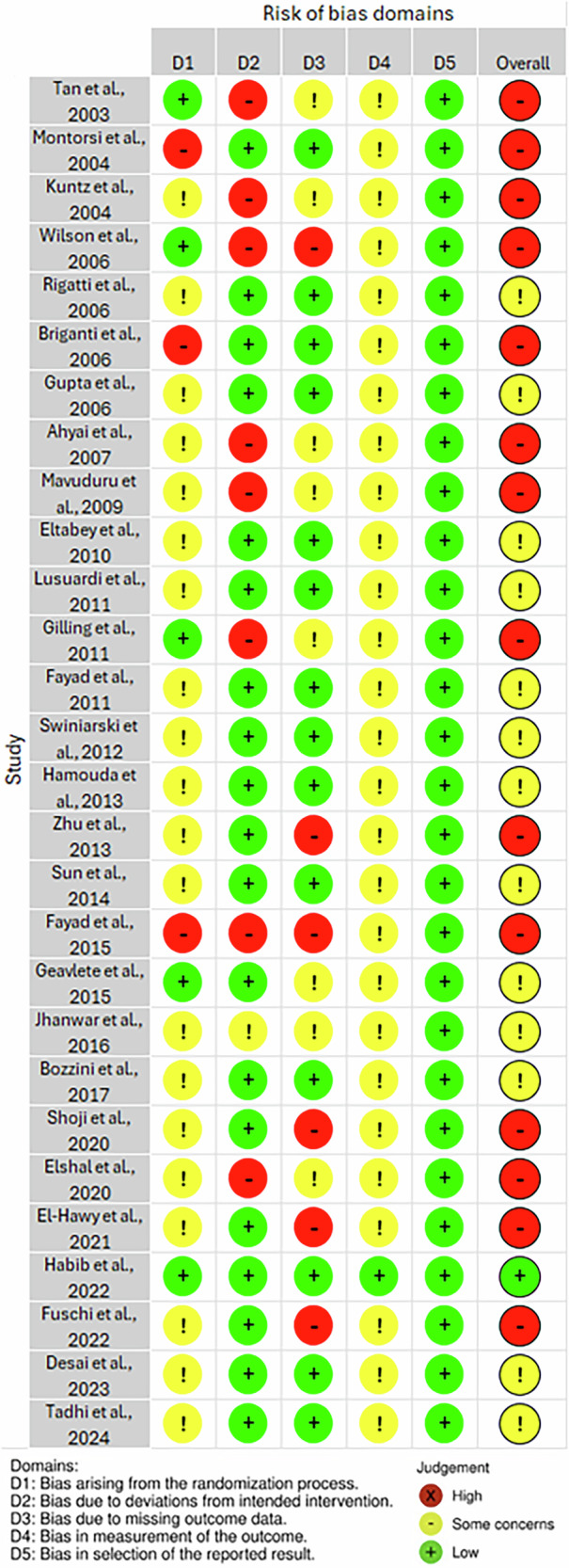
Risk of bias assessment. Risk of bias assessment using Cochrane’s RoB2 tool.

The risk of bias for the included studies was assessed using the Cochrane Risk of Bias 2 (RoB 2) tool (Fig. 2). The majority of included studies exhibited either a high risk of bias or some concerns Out of the 28 studies, 14 studies had a high risk of bias, 13 studies had some concern, with only 1 study having a low risk of bias. Authors provided or reported no blinding, and there was significant drop-out in many studies. Patients were also aware of their procedure type and expectations may have influenced the answers of their questionnaires and therefore the outcomes that relied on self-reports.

### Functional outcomes

#### IPSS

**Fig. 3:**
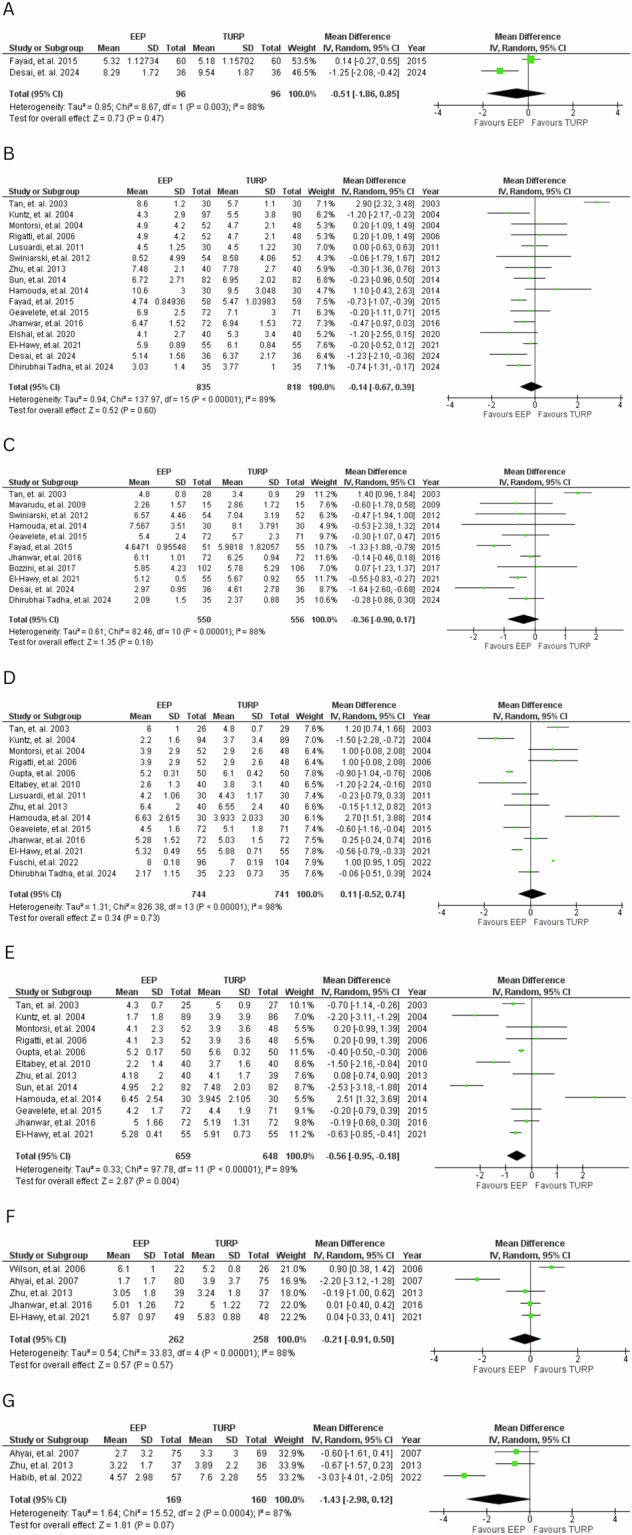
IPSS scores. Forest plot comparing IPSS at various time points for EEP versus TURP. **A**—1 week, **B**—1 month, **C**—3 months, **D**—6 months, **E**—12 months, **F**—24 months, **G**—36 months.

12 distinct time points were identified with data for IPSS, with 7 of these having more than one study to compare (Fig. 3). Although most pooled estimates slightly favoured EEP over TURP, significant differences were only observed at 12 months post-operation (MD = −0.56, 95% CI: −0.95 to −0.18, *P* = 0.004). At other time points, the differences were not statistically significant.

#### Qmax

**Fig. 4:**
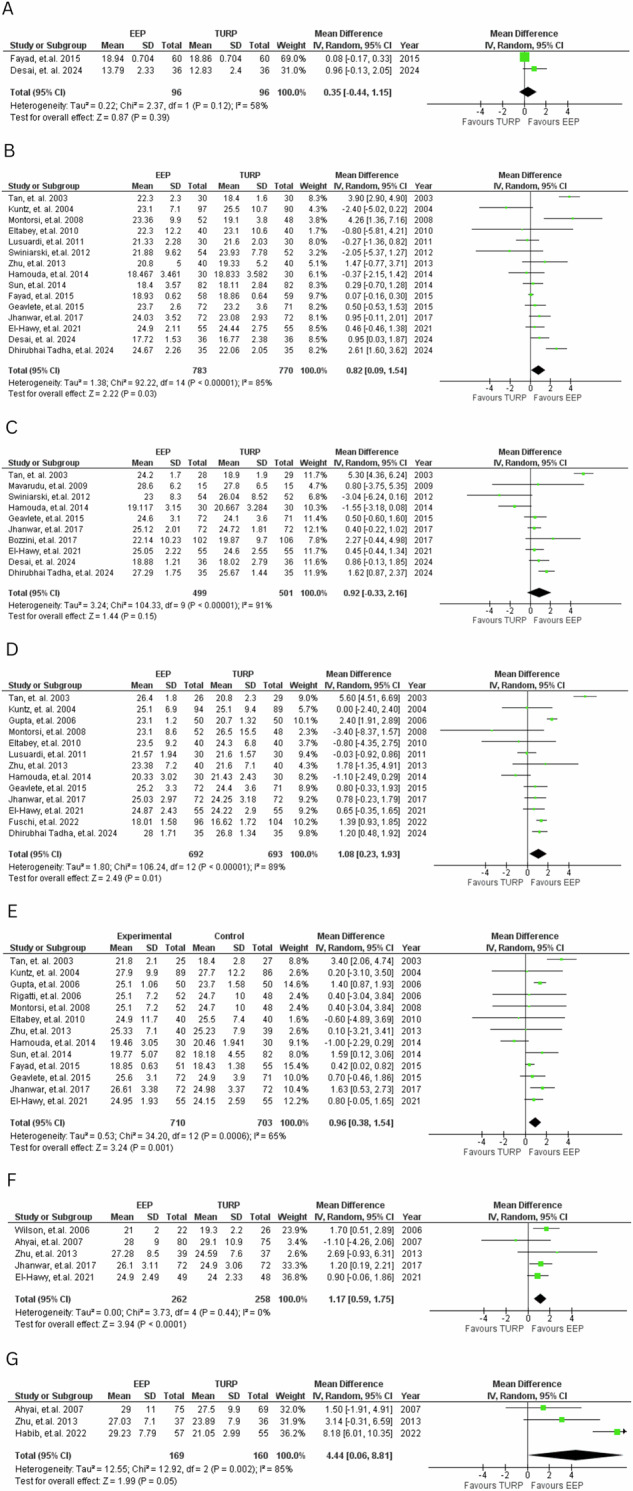
Qmax scores. Forest plot comparing Qmax at various time points for EEP versus TURP. **A**—1 week, **B**—1 month, **C**—3 months, **D**—6 months, **E**—12 months, **F**—24 months, **G**—36 months.

7-time points with studies to compare were identified, as shown in Fig. 4. EEP consistently showed trends towards improved Qmax across multiple post-operative time points. Statistically significant benefits favouring EEP were observed at 1 month (MD = 0.82, 95% CI: 0.09 to 1.54; *P* = 0.03), 6 months (MD = 1.08, 95% CI: 0.23 to 1.93; *P* = 0.01), 12 months (MD = 0.96, 95% CI: 0.38 to 1.54; *P* = 0.001), and 24 months (MD = 1.17, 95% CI: 0.59 to 1.75; *P* < 0.001) post-operation.

#### QOL

**Fig. 5:**
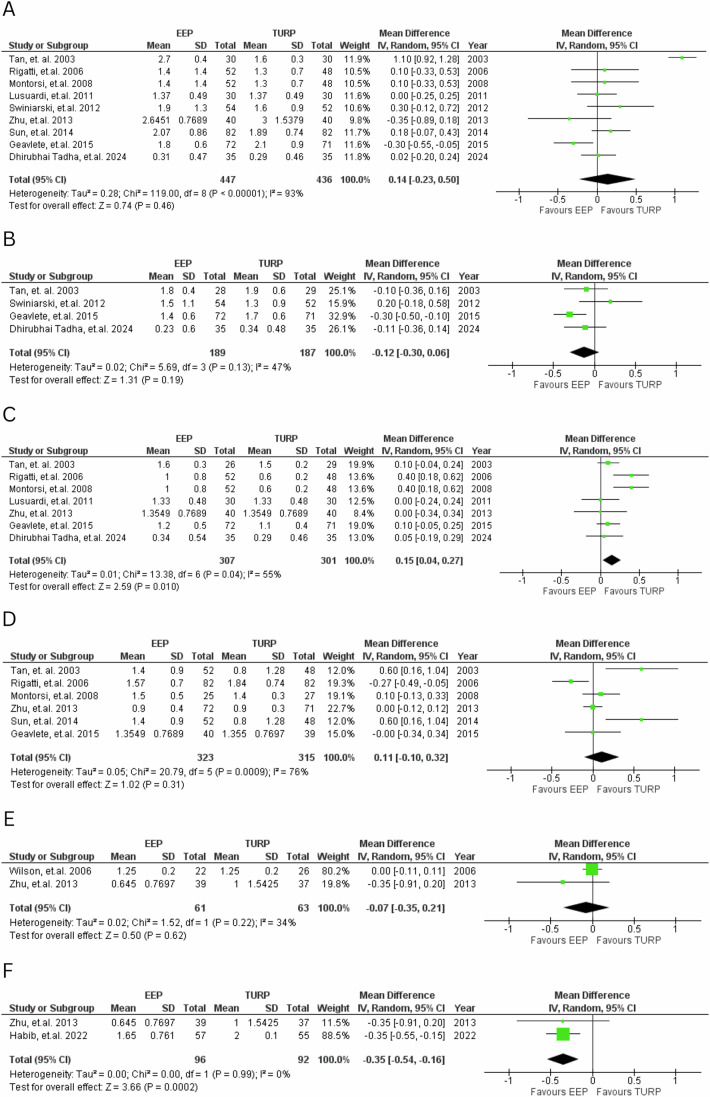
QOL scores. Forest plot comparing QOL scores at various time points for EEP versus TURP. **A**—1 month, **B**—3 months, **C**—6 months, **D**—12 months, **E**—24 months, **F**—36 months.

Significant improvements in QOL initially favoured TURP at 6 months (MD = 0.15, 95% CI: 0.04 to 0.27; *P* = 0.01), whereas EEP demonstrated a clear benefit at 36 months (MD = −0.35, 95% CI: −0.54 to −0.16; *P* < 0.001). Results are demonstrated in Fig. 5.

### PVR values

**Fig. 6:**
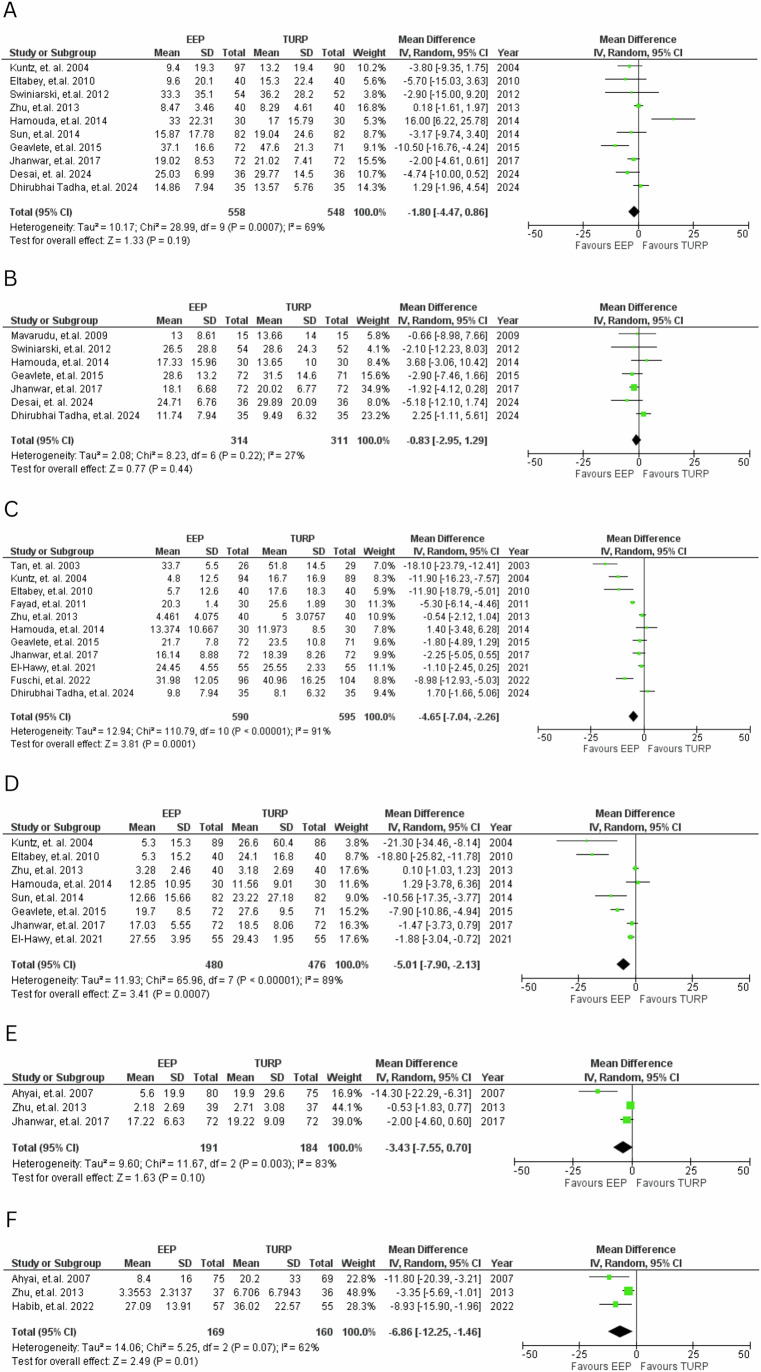
PVR values. Forest plot comparing PVR values at various time points for EEP versus TURP. **A**—1 month, **B**—3 months, **C**—6 months, **D**—12 months, **E**—24 months, **F**—36 months.

EEP significantly reduced PVR volumes at 6 months (MD = −4.65, 95% CI: −7.04 to −2.26; *P* < 0.001), 12 months (MD = −5.01, 95% CI: −7.90 to −2.13; *P* < 0.001), and 36 months (MD = −6.86, 95% CI: −12.25 to −1.46; *P* = 0.01), seen in Fig. 6.

### IIEF-5 score

**Fig. 7:**
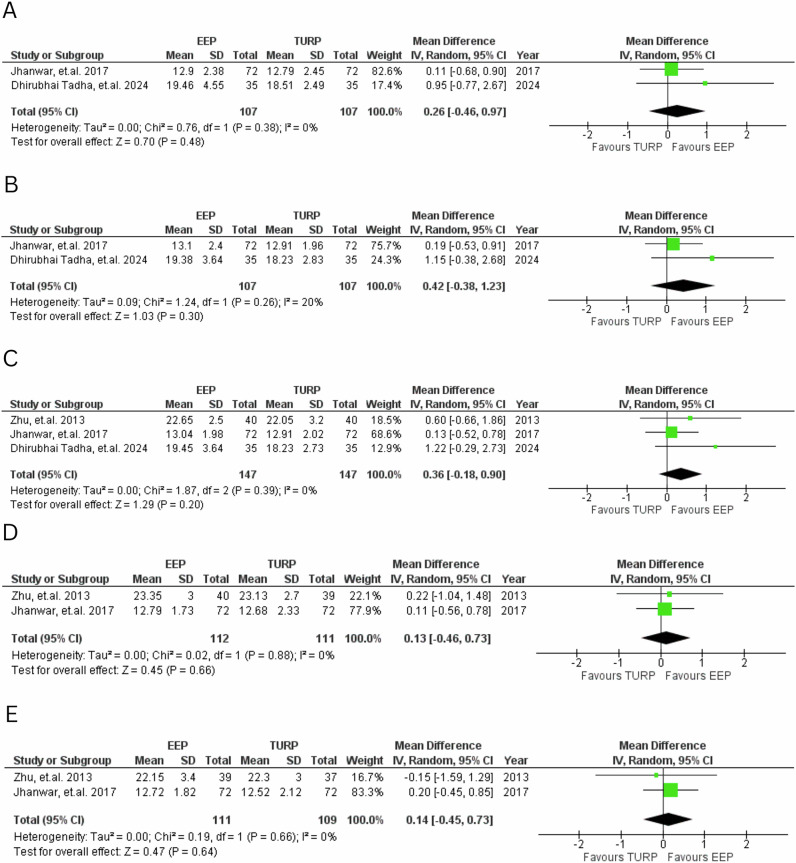
IIEF-5 scores. Forest plot comparing IIEF-5 at various time points for EEP versus TURP. **A**—1 month, **B**—3 months, **C**—6 months, **D**—12 months, **E**—24 months.

3 studies evaluated sexual function outcomes across 5 different time points (Fig. 7). Although EEP was favoured over TURP (MD = 1.13 to 0.42), none of these differences reached statistical significance at any time point. Heterogeneity was low to negligible across all analyses.

### Hospital stay duration

**Fig. 8:**
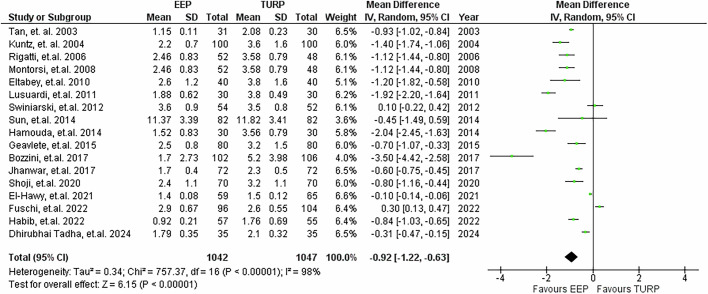
Hospital stay duration. Forest plot illustrating the mean difference of hospital stay duration between EEP and TURP.

17 studies reported on hospital stay, highlighted in Fig. 8. Compared with TURP, EEP significantly reduced length of stay by a mean of 0.92 days (95% CI: −1.22 to −0.63; *P* < 0.001).

### Catheterisation time

**Fig. 9:**
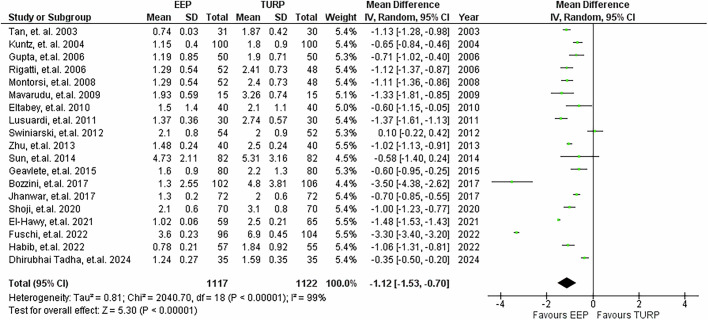
Catheterisation time. Forest plot illustrating the mean difference of catheterisation time between EEP and TURP.

19 studies reported on catheterisation time, illustrated in Fig. 9. EEP significantly reduced catheter duration compared with TURP by approximately 1.12 days (95% CI: −1.53 to −0.70, *P* < 0.001).

### Transfusion rates

**Fig. 10:**
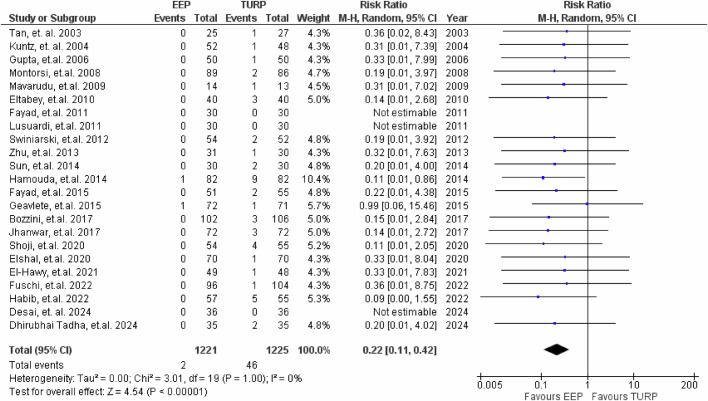
Transfusion rates. Forest plot illustrating the relative risk of immediate post-operative transfusion between EEP and TURP.

23 studies reported transfusion rates, seen in Fig. 10. EEP was associated with a significantly lower risk of requiring transfusions, (RR = 0.22, 95% CI: 0.11 to 0.42; *P* < 0.001), corresponding to a 78% reduction compared to TURP.

### Infection rates

**Fig. 11:**
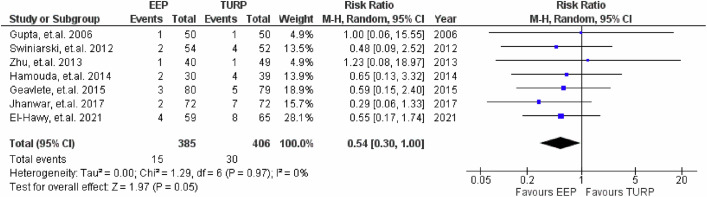
Infection rates. Forest plot illustrating the relative risk of immediate post-operative infection between EEP and TURP.

7 studies assessed immediate post-operative infection rates, with results shown in Fig. 11. The pooled analysis indicated that EEP was associated with a reduction in the risk of infection compared to TURP (RR = 0.56, 95% CI: 0.30 to 1.03; *P* = 0.06), but this did not reach statistical significance. There was also an absence of heterogeneity.

### Stricture rates

**Fig. 12:**
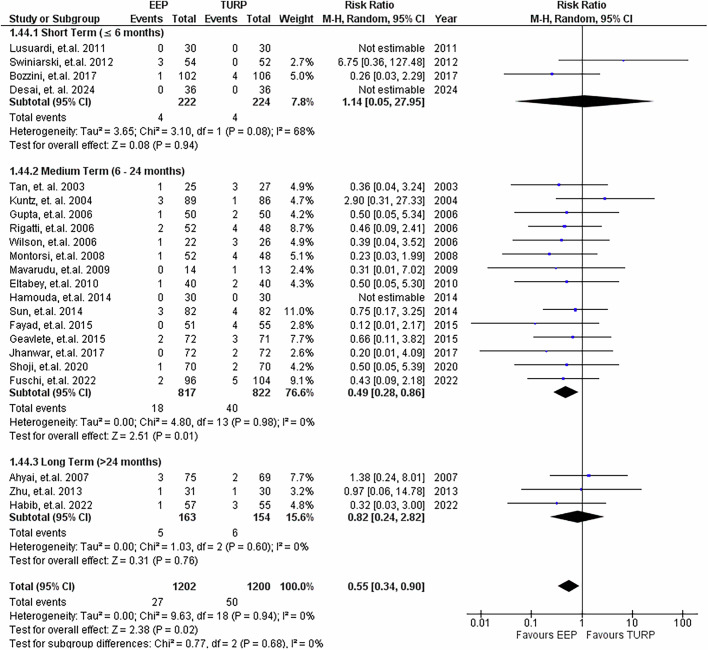
Stricture rates. Forest plot illustrating the relative risk of developing strictures post-procedure between EEP and TURP.

22 studies reported on stricture rates post-procedure, demonstrated in Fig. 12. Pooled analysis favoured EEP (RR = 0.55, 95% CI: 0.34 to 0.90; *P* = 0.02), indicating a reduction in the odds of developing urethral strictures relative to TURP, with heterogeneity being absent. Subgroup analysis demonstrated that this was only statistically significant in the medium term (6–24 months).

### Bladder neck contracture rates

**Fig. 13:**
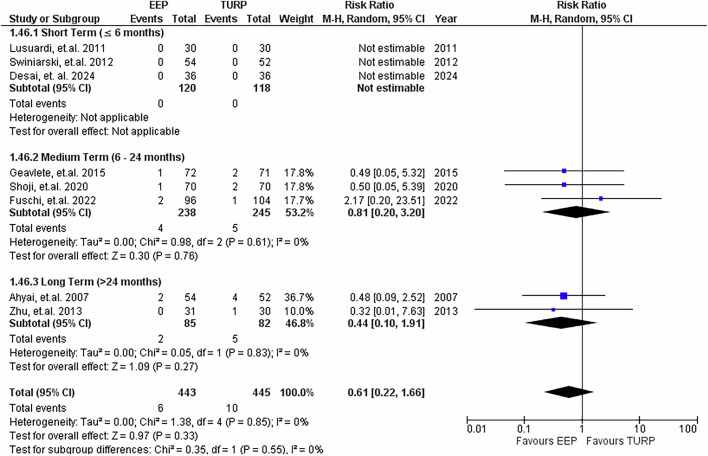
Bladder neck contracture rates. Forest plot illustrating the relative risk of developing bladder neck contractures post-procedure between EEP and TURP.

8 studies assessed bladder neck contractures, as seen in Fig. 13. The pooled RR was 0.61 (95% CI: 0.22 to 1.66; *P* = 0.55), suggesting no significant difference between EEP and TURP. Heterogeneity was absent. Subgroup analyses based on follow-up duration were performed. Due to insufficient data, the risk ratio for bladder neck contractures in the short-term follow-up period could not be calculated. In the medium and long term, there was no significant difference in BNC rates.

### Incontinence

**Fig. 14:**
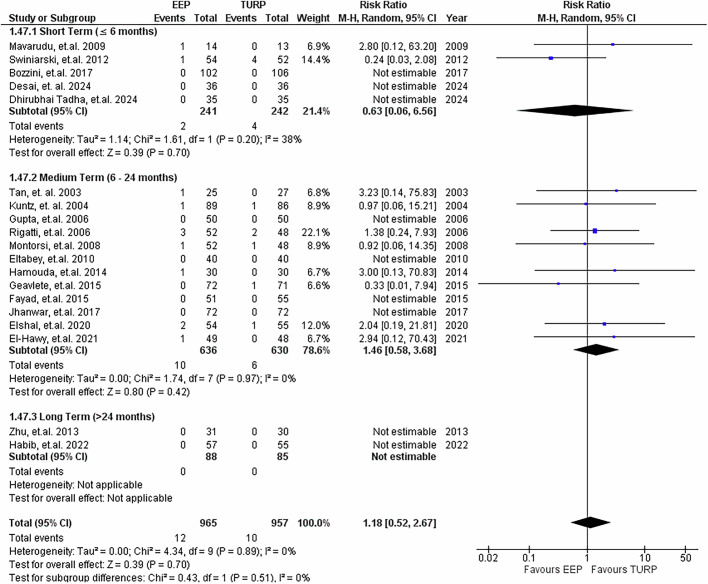
Incontinence rates. Forest plot illustrating the relative risk of developing incontinence post-procedure between EEP and TURP.

19 studies provided data on post-operative incontinence, as per Fig. 14. In there were no significant differences between either group at all three follow-up time points. Long-term data (>24 months) were not estimable because neither group reported any incontinence events in the relevant studies. When the data were pooled across all time frames, the overall RR was 1.18 (95% CI: 0.52 to 2.67; *P* = 0.70), again showing no statistically significant difference.

### Reoperation rate due to BPH regrowth

**Fig. 15:**
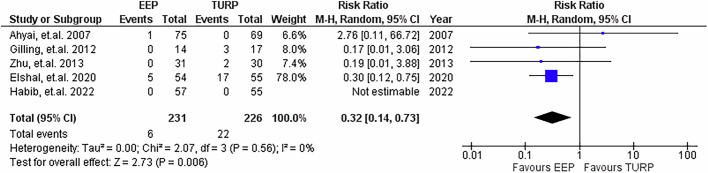
Re-operation rate due to BPH regrowth. Forest plot illustrating the relative risk of undergoing re-operation for BPH regrowth post-procedure between EEP and TURP.

5 studies provided data on reoperation rates specifically to recurrence of BPH, with a minimum follow-up duration of 3 years, demonstrated in Fig. 15. Pooled analysis demonstrated a significantly lower rate following EEP, with RR being 0.32 (95% CI: 0.14 to 0.73, *P* = 0.006). Heterogeneity was absent.

## Discussion

Our meta-analysis of 28 randomised controlled trials (RCTs) demonstrates that EEP is at least non-inferior, and in many instances, superior to TURP. Although each energy source may differ in operative parameters, learning curve and outcomes, we pooled these techniques under the broader EEP category, as complete anatomical dissection of the prostatic adenoma along the surgical capsule remains primary determinant of clinical key outcomes [33, 34]. Future analyses that focus on each energy source independently would require more consistent reporting of device type and settings across studies.

One of the most clinically significant findings of this analysis is improved perioperative outcomes associated with EEP. Lower transfusion requirements, shorter catheterisation time, and shorter hospital stays likely stem from the direct coagulation of capsular vessels and efficient haemostasis inherent to an anatomical enucleation approach [33, 34]. Netsch et. al. [35] has likewise highlighted that EEP is feasible and efficacious in patients on antiplatelets or anticoagulation, although the data remains sparse.

Our pooled analysis suggests that longer-term adverse events such as incontinence and bladder neck contracture (BNC) rates did not differ significantly between the two procedures. Notably, stricture rates were reduced in the EEP group. Re-treatment rates specifically for BPH were not widely reported but also favoured EEP. These results imply that EEP may provide more durable symptom control, and a reduced likelihood of subsequent surgical intervention compared with TURP.

Early enucleation techniques, as described by Gilling [36], adopt a multi-lobe approach, followed by mechanical morcellation. More recently, en-bloc variants strive to remove the adenoma in a single piece [37, 38]. While no definitive trial has compared these approaches head-to-head, both share the aim of achieving a complete adenoma removal that mimics the principles of open simple prostatectomy. By fully removing the hyperplastic tissue, EEP maximises bladder outlet obstruction relief and theoretically lowers the risk of re-growth or secondary procedures [33].

While functional outcomes remain pivotal in routine clinical practice, our findings underscore the importance of adverse outcomes and re-treatment in shaping patient counselling and long-term follow-up.

Earlier meta-analyses comparing EEP and TURP had fewer included trials or narrower inclusion criteria, sometimes focusing exclusively on a single energy source or specific enucleation method. Both Tan et. al. [39] and Yin et. al. [40] restricted their evaluations to holmium laser versus TURP alone. By broadening the scope to incorporate various energy sources and follow-up durations, our meta-analysis of 28 studies incorporates one of the largest sets of RCT data currently available.

Wroclawski et. al. [34] conducted a meta-analysis encompassing multiple forms of endoscopic treatments for BPH and concluded that EEP offered superior Qmax and IPSS improvements, reduced catheterisation times and lower transfusion rates versus non-enucleation approaches. These included TURP, plasma kinetic resection and vaporisation techniques. Similarly, Chen et. al. [33] reported at least equivalent medium-term (>12 months) benefits in Qmax, PVR and IPSS scores for EEP versus TURP, alongside improved peri-operative parameters such as haemoglobin loss, catheterisation times and hospital stay. Longer-term outcomes (>3 years) were addressed in Mozorov et. al.’s [2] meta-analysis, which found a significantly lower reoperation rate with EEP compared to TURP, in addition to superior Qmax and IPSS outcomes favouring EEP.

Compared to prior analyses, our study integrates findings from laser-based and electrocautery-based EEP and evaluates both peri-operative and long-term outcomes. This extends the conclusion that EEP confers a consistent trend towards better safety profiles and enduring symptom relief over TURP.

Surgeon expertise and institutional resources often guide which energy source is used for EEP. Holmium and thulium fibre lasers allow concurrent bladder stone management, an appealing option where stone disease coexists. Many modern urology departments already have laser systems for stone treatment, facilitating laser-based EEP. Newer pulsed thulium:YAG lasers also have recently emerged, but require further study to clarify their role in both enucleation and stone treatment [41, 42], Meanwhile, bipolar electrocautery can be more readily available and less costly. Prior research shows no clear superiority of one energy modality over another once the surgeon has mastered the enucleation technique [43, 44].

Thus, the decision primarily hinges on logistics, accessibility, and surgeon familiarity rather than a proven efficacy difference.

For practising urologists, EEP offers multiple benefits over TURP. The first being lower bleeding risk, making it particularly beneficial for patients with coagulopathies or those on anticoagulant or antiplatelet therapies. Secondly, sustained functional improvements as evidenced by consistently favourable Qmax, IPSS and PVR outcomes. Lower re-treatment rates and significant stricture reduction compared to TURP also suggest long-term durability. Finally, rates of incontinence, BNC and infection are broadly similar between EEP and TURP, minimising concerns about elevated complications.

However, many surgeons still prefer TURP due to its familiarity, potentially shorter operative time and traditional teaching patterns in residency and fellowship programs [45]. Moreover, EEP’s learning curve can be significant. A meta-analysis from Enikeev et. al. [46] demonstrates a steep learning curve, with a plateau after 30-50 cases. Cases early on during training have a higher rate of complications, including stress urinary incontinence. This is contrasted to TURP, where the learning curve is approximately 10 cases [47]. Institutions must consider whether they possess the resources and training pathways to adopt EEP effectively.

### Limitations

Several limitations should be recognised. Firstly, the risk of bias was high or of some concern in nearly all the included studies, commonly due to inadequate blinding and incomplete follow-up. The potential placebo effect could also influence self-reported measures such as IPSS or QOL since patients were generally aware of which procedure they received. Secondly, there was notable heterogeneity in outcomes—a reflection of differences in patient characteristics (prostate volume, co-morbidities, anticoagulation use), surgeon experience and variations in enucleation technique (multi-lobe versus en-bloc). Such heterogeneity may have resulted in over- or under-estimation of true effect sizes. Thirdly, we did not perform a cost-effectiveness analysis, as most included studies did not report cost-related outcomes. Future research assessing resource utilisation and financial impact would better inform clinical decision-making. Fourthly, although we recorded prostate sizes, insufficient data prevented formal subgroup analyses by volume or by specific surgical technique methods. Additionally, inconsistent follow-up intervals and variable outcome reporting limited the potential for uniform subgroup analyses. Although we categorised short-, medium- and long-term studies, many trials assessed endpoints at different time points or did not report certain outcomes such as sexual function. Finally, our systematic review focused on RCTs in English and it is possible that unpublished or non-English trials with differing results were missed.

## Conclusion

Various forms of EEP compare favourably with TURP for managing BPH, offering significant advantages in peri-operative bleeding control, short-term recovery, and a more durable relief of obstruction. Our findings indicate that an anatomical enucleation-based approach—regardless of energy source—can achieve at least comparable and often superior outcomes compared with TURP. Although further high-quality trials controlling for technique and surgeon proficiency are needed to clarify the impact of enucleation completeness versus energy source on long-term outcomes, the choice of energy source can be guided by institutional resources and surgeon preference.

## Supplementary information

Search Strategy

Supplementary Table 1
