# Supplementary material for: Endoscopic enucleation of the prostate versus transurethral resection of the prostate for benign prostatic hyperplasia: a systematic review and meta-analysis
Source: Prostate Cancer Prostatic Dis. 2025 May 10;28(4):845–58. doi: 10.1038/s41391-025-00970-z (PMC12643921; doi:10.1038/s41391-025-00970-z)
Supplement: Supplementary file 1 — Search Strategy [file 41391_2025_970_MOESM1_ESM.docx]

Appendix 1

PubMed

7 #1 AND #2 AND #3 AND #6

6 #4 NOT #5

5 animals [mh] NOT humans [mh]

4 randomized controlled trial [pt] OR controlled clinical trial [pt] OR randomized [tiab] OR placebo [tiab] OR drug therapy [sh] OR randomly [tiab] OR trial [tiab] OR groups [tiab]

3 (TURP OR resect*)

2 (endoscop* AND enucleat*) OR (endoscopic AND enucleation AND of AND the AND prostate OR eep) OR (anatomical AND endoscopic AND enucleation AND of AND the AND prostate OR aeep) OR (Holmium OR HoLEP) OR (Thulium OR ThuLEP) OR (Bipolar OR BipoLEP) OR (plasmakinetic OR PkEP) OR (diode OR DiLEP) OR (photo* AND vapori* OR photovapori* OR photo-vapori* OR greenlight OR PVP)

1 ("Prostatic Hyperplasia"[MeSH] OR "Prostatic Neoplasms"[MeSH] OR "Prostate"[MeSH] OR "prostat* hyper*" OR "BPH")

Embase <1996 to 2024 May 15>

1 exp prostatic hyperplasia/ 36665

2 exp prostatic neoplasm/ 286434

3 exp prostate/ 50537

4 exp endoscopic surgery/ and exp enucleation/ 841

5 "endoscop* enucleat*".mp. 355

6 ("endoscopic enucleation of the prostate" or EEP).mp. 1218

7 ("anatomical endoscopic enucleation of the prostate" or AEEP).mp. 52

8 (Holmium or HoLEP).mp. 10527

9 (Thulium or ThuLEP).mp. 3453

10 (Bipolar or BipoLEP).mp. 128139

11 (plasmakinetic or PkEP).mp. 411

12 (diode or DiLEP).mp. 51107

13 ("photo* vapori*" or "photovapori*" or "photo-vapori*" or greenlight or PVP).mp. 13981

14 (TURP or resect*).mp. 695386

15 1 or 2 or 3 337426

16 4 or 5 or 6 or 7 or 8 or 9 or 10 or 11 or 12 or 13 206253

17 14 and 15 and 16 3090

18 exp randomized controlled trial/ 777180

19 controlled clinical trial/ 427641

20 random$.ti,ab. 1927962

21 randomization/ 90968

22 intermethod comparison/ 296814

23 placebo.ti,ab. 327609

24 (compare or compared or comparison).ti,ab. 7145374

25 ((evaluated or evaluate or evaluating or assessed or assess) and (compare or compared or comparing or comparison)).mp. [mp=title, abstract, heading word, drug trade name, original title, device manufacturer, drug manufacturer, device trade name, keyword heading word, floating subheading word, candidate term word] 2985861

26 (open adj label).ti,ab. 113825

27 ((double or single or doubly or singly) adj (blind or blinded or blindly)).ti,ab. 233515

28 double blind procedure/ 192405

29 parallel group$1.ti,ab. 31314

30 (crossover or cross over).ti,ab. 107817

31 ((assign$ or match or matched or allocation) adj5 (alternate or group$1 or intervention$1 or patient$1 or subject$1 or participant$1)).ti,ab. 400024

32 (assigned or allocated).ti,ab. 470913

33 (controlled adj7 (study or design or trial)).ti,ab. 439884

34 (volunteer or volunteers).ti,ab. 238312

35 human experiment/ 522956

36 trial.ti. 391958

37 18 or 19 or 20 or 21 or 22 or 23 or 24 or 25 or 26 or 27 or 28 or 29 or 30 or 31 or 32 or 33 or 34 or 35 or 36 9390267

38 cross-sectional study/de not (exp randomized controlled trial/ or controlled clinical trial/ or controlled study/ or randomi?ed controlled.ti,ab. or control group$1.ti,ab.) 0

39 ((case adj control$).mp. and random$.ti,ab.) not randomi?ed controlled.ti,ab. [mp=title, abstract, heading word, drug trade name, original title, device manufacturer, drug manufacturer, device trade name, keyword heading word, floating subheading word, candidate term word] 26019

40 (nonrandom$ not random$).ti,ab. 16867

41 (systematic review not (trial or study)).ti,ab. 177602

42 "random field$".ti,ab. 2989

43 (random cluster adj3 sampl$).ti,ab. 1600

44 (review.ab. and review.pt.) not trial.ti. 1160929

45 "we searched".ab. and (review.ti. or review.pt.) 52556

46 "update review".ab. 131

47 (databases adj4 searched).ab. 67846

48 (rat or rats or mouse or mice or swine or porcine or murine or sheep or lambs or pigs or piglets or rabbit or rabbits or cat or cats or dog or dogs or cattle or bovine or monkey or monkeys or trout or marmoset$1).ti. and animal experiment/ 874353

49 animal experiment/ not (human experiment/ or human/) 1819816

50 38 or 39 or 40 or 41 or 42 or 43 or 44 or 45 or 46 or 47 or 48 or 49 3239326

51 37 not 50 8355529

52 17 and 51 1584

Ovid MEDLINE® including Daily update <1996-current>

1 exp Prostatic Hyperplasia/ 16344

2 exp Prostatic Neoplasms/ 130243

3 exp Prostate/ 29336

4 (endoscop* and enucleat*).tw. 559

5 (endoscopic enucleation of the prostate or EEP).tw. 651

6 (anatomical endoscopic enucleation of the prostate or AEEP).tw. 25

7 (Holmium or HoLEP).tw. 2507

8 (Thulium or ThuLEP).tw. 922

9 (Bipolar or BipoLEP).tw. 55073

10 (plasmakinetic or PkEP).tw. 217

11 (diode or DiLEP).tw. 17137

12 (photo* vapori* or photovapori* or photo-vapori* or greenlight or PVP).tw. 6963

13 (TURP or resect*).tw. 298308

14 randomized controlled trial.pt. 518141

15 controlled clinical trial.pt. 50227

16 randomized.ab. 513562

17 placebo.ab. 183138

18 drug therapy.fs. 2055153

19 randomly.ab. 324761

20 trial.ab. 546476

21 groups.ab. 1909219

22 14 or 15 or 16 or 17 or 18 or 19 or 20 or 21 4354922

23 exp animals/ not humans.sh. 3004839

24 22 not 23 3782357

25 1 or 2 or 3 151546

26 4 or 5 or 6 or 7 or 8 or 9 or 10 or 11 or 12 82951

27 13 and 24 and 25 and 26 492

Scopus

( TITLE-ABS-KEY ( "prostatic hyperplasia" ) OR TITLE-ABS-KEY ( "prostatic neoplasm" ) OR TITLE-ABS-KEY ( "prostate" ) ) AND ( TITLE-ABS-KEY ( endoscop* AND enucleat* ) OR TITLE-ABS-KEY ( endoscopic AND enucleation AND of AND the AND prostate OR eep ) OR TITLE-ABS-KEY ( anatomical AND endoscopic AND enuclation AND of AND the AND prostate OR aeep ) OR TITLE-ABS-KEY ( holmium OR holep ) OR TITLE-ABS-KEY ( thulium OR thulep ) OR TITLE-ABS-KEY ( bipolar OR bipolep ) OR TITLE-ABS-KEY ( plasmakinetic OR pkep ) OR TITLE-ABS-KEY ( diode OR dilep ) OR TITLE-ABS-KEY ( photo* AND vapori* OR photovapori* OR photo-vapori* OR greenlight OR pvp ) ) AND ( TITLE-ABS-KEY ( turp OR resect* ) ) AND ( INDEXTERMS ( "clinical trials" OR "clinical trials as a topic" OR "randomized controlled trial" OR "Randomized Controlled Trials as Topic" OR "controlled clinical trial" OR "Controlled Clinical Trials" OR "random allocation" OR "Double-Blind Method" OR "Single-Blind Method" OR "Cross-Over Studies" OR "Placebos" OR "multicenter study" OR "double blind procedure" OR "single blind procedure" OR "crossover procedure" OR "clinical trial" OR "controlled study" OR "randomization" OR "placebo" ) ) OR ( TITLE-ABS-KEY ( ( "clinical trials" OR "clinical trials as a topic" OR "randomized controlled trial" OR "Randomized Controlled Trials as Topic" OR "controlled clinical trial" OR "Controlled Clinical Trials as Topic" OR "random allocation" OR "randomly allocated" OR "allocated randomly" OR "Double-Blind Method" OR "Single-Blind Method" OR "Cross-Over Studies" OR "Placebos" OR "cross-over trial" OR "single blind" OR "double blind" OR "factorial design" OR "factorial trial" ) ) ) OR ( TITLE-ABS ( clinical AND trial* OR trial* OR rct* OR random* OR blind* ) )

Web of Science

1: TS=("prostat*") Date Run: Thu May 16 2024 15:28:58 GMT+1000 (Australian Eastern Standard Time) Results: 399341

2: (TS=(endoscop* AND enucleat*) OR TS=(endoscopic AND enucleation AND of AND the AND prostate OR eep) OR TS=(anatomical AND endoscopic AND enucleation AND of AND the AND prostate OR aeep) OR TS=(holmium OR holep) OR TS=(thulium OR thulep) OR TS=(bipolar OR bipolep) OR TS=(plasmakinetic OR pkep) OR TS=(diode OR dilep) OR TS=(photo* AND vapori* OR photovapori* OR photo-vapori* OR greenlight OR pvp)) Date Run: Thu May 16 2024 15:29:13 GMT+1000 (Australian Eastern Standard Time) Results: 523258

3: TS=(turp OR resect*) Date Run: Thu May 16 2024 15:29:21 GMT+1000 (Australian Eastern Standard Time) Results: 463952

4: TS=(randomised OR randomized OR randomisation OR randomisation OR placebo* OR (random* AND (allocat* OR assign*) ) OR (blind* AND (single OR double OR treble OR triple) )) NOT TS=(animal or animals or pisces or fish or fishes or catfish or catfishes or sheatfish or silurus or arius or heteropneustes or clarias or gariepinus or fathead minnow or fathead minnows or pimephales or promelas or cichlidae or trout or trouts or char or chars or salvelinus or salmo or oncorhynchus or guppy or guppies or millionfish or poecilia or goldfish or goldfishes or carassius or auratus or mullet or mullets or mugil or curema or shark or sharks or cod or cods or gadus or morhua or carp or carps or cyprinus or carpio or killifish or eel or eels or anguilla or zander or sander or lucioperca or stizostedion or turbot or turbots or psetta or flatfish or flatfishes or plaice or pleuronectes or platessa or tilapia or tilapias or oreochromis or sarotherodon or common sole or dover sole or solea or zebrafish or zebrafishes or danio or rerio or seabass or dicentrarchus or labrax or morone or lamprey or lampreys or petromyzon or pumpkinseed or pumpkinseeds or lepomis or gibbosus or herring or clupea or harengus or amphibia or amphibian or amphibians or anura or salientia or frog or frogs or rana or toad or toads or bufo or xenopus or laevis or bombina or epidalea or calamita or salamander or salamanders or newt or newts or triturus or reptilia or reptile or reptiles or bearded dragon or pogona or vitticeps or iguana or iguanas or lizard or lizards or anguis fragilis or turtle or turtles or snakes or snake or aves or bird or birds or quail or quails or coturnix or bobwhite or colinus or virginianus or poultry or poultries or fowl or fowls or chicken or chickens or gallus or zebra finch or taeniopygia or guttata or canary or canaries or serinus or canaria or parakeet or parakeets or grasskeet or parrot or parrots or psittacine or psittacines or shelduck or tadorna or goose or geese or branta or leucopsis or woodlark or lullula or flycatcher or ficedula or hypoleuca or dove or doves or geopelia or cuneata or duck or ducks or greylag or graylag or anser or harrier or circus pygargus or red knot or great knot or calidris or canutus or godwit or limosa or lapponica or meleagris or gallopavo or jackdaw or corvus or monedula or ruff or philomachus or pugnax or lapwing or peewit or plover or vanellus or swan or cygnus or columbianus or bewickii or gull or chroicocephalus or ridibundus or albifrons or great tit or parus or aythya or fuligula or streptopelia or risoria or spoonbill or platalea or leucorodia or blackbird or turdus or merula or blue tit or cyanistes or pigeon or pigeons or columba or pintail or anas or starling or sturnus or owl or athene noctua or pochard or ferina or cockatiel or nymphicus or hollandicus or skylark or alauda or tern or sterna or teal or crecca or oystercatcher or haematopus or ostralegus or shrew or shrews or sorex or araneus or crocidura or russula or european mole or talpa or chiroptera or bat or bats or eptesicus or serotinus or myotis or dasycneme or daubentonii or pipistrelle or pipistrellus or cat or cats or felis or catus or feline or dog or dogs or canis or canine or canines or otter or otters or lutra or badger or badgers or meles or fitchew or fitch or foumart or foulmart or ferrets or ferret or polecat or polecats or mustela or putorius or weasel or weasels or fox or foxes or vulpes or common seal or phoca or vitulina or grey seal or halichoerus or horse or horses or equus or equine or equidae or donkey or donkeys or mule or mules or pig or pigs or swine or swines or hog or hogs or boar or boars or porcine or piglet or piglets or sus or scrofa or llama or llamas or lama or glama or deer or deers or cervus or elaphus or cow or cows or bos taurus or bos indicus or bovine or bull or bulls or cattle or bison or bisons or sheep or sheeps or ovis aries or ovine or lamb or lambs or mouflon or mouflons or goat or goats or capra or caprine or chamois or rupicapra or leporidae or lagomorpha or lagomorph or rabbit or rabbits or oryctolagus or cuniculus or laprine or hares or lepus or rodentia or rodent or rodents or murinae or mouse or mice or mus or musculus or murine or woodmouse or apodemus or rat or rats or rattus or norvegicus or guinea pig or guinea pigs or cavia or porcellus or hamster or hamsters or mesocricetus or cricetulus or cricetus or gerbil or gerbils or jird or jirds or meriones or unguiculatus or jerboa or jerboas or jaculus or chinchilla or chinchillas or beaver or beavers or castor fiber or castor canadensis or sciuridae or squirrel or squirrels or sciurus or chipmunk or chipmunks or marmot or marmots or marmota or suslik or susliks or spermophilus or cynomys or cottonrat or cottonrats or sigmodon or vole or voles or microtus or myodes or glareolus or primate or primates or prosimian or prosimians or lemur or lemurs or lemuridae or loris or bush baby or bush babies or bushbaby or bushbabies or galago or galagos or anthropoidea or anthropoids or simian or simians or monkey or monkeys or marmoset or marmosets or callithrix or cebuella or tamarin or tamarins or saguinus or leontopithecus or squirrel monkey or squirrel monkeys or saimiri or night monkey or night monkeys or owl monkey or owl monkeys or douroucoulis or aotus or spider monkey or spider monkeys or ateles or baboon or baboons or papio or rhesus monkey or macaque or macaca or mulatta or cynomolgus or fascicularis or green monkey or green monkeys or chlorocebus or vervet or vervets or pygerythrus or hominoidea or ape or apes or hylobatidae or gibbon or gibbons or siamang or siamangs or nomascus or symphalangus or hominidae or orangutan or orangutans or pongo or chimpanzee or chimpanzees or pan troglodytes or bonobo or bonobos or pan paniscus or gorilla or gorillas or troglodytes) Date Run: Thu May 16 2024 15:29:54 GMT+1000 (Australian Eastern Standard Time) Results: 1430015

5: #4 AND #3 AND #2 AND #1 Date Run: Thu May 16 2024 15:30:09 GMT+1000 (Australian Eastern Standard Time) Results: 1022
