# Supplementary material for: Endoscopic enucleation of the prostate versus transurethral resection of the prostate for benign prostatic hyperplasia: a systematic review and meta-analysis
Source: Prostate Cancer Prostatic Dis. 2025 May 10;28(4):845–58. doi: 10.1038/s41391-025-00970-z (PMC12643921; doi:10.1038/s41391-025-00970-z)
Supplement: Supplementary file 2 — Supplementary Table 1 [file 41391_2025_970_MOESM2_ESM.docx]

| **Authors, Year** | **Article Name** | **Follow-Up Duration** | **Groups** | **Number of Patients Randomised** | **Energy Source** | **Number of Patients Followed Up** | **Mean Age (Years)** | **Prostate Volume** | **Catheterisation Time (Days)** | **Hospital Stay Duration (Days)** | **IPSS – Baseline** | **IPSS – Final** | **QOL Score – Baseline** | **QOL Score –Final** | **Qmax – Baseline (mL/s)** | **Qmax – Final (mL/s)** | **PVR – Baseline (mL)** | **PVR – Final (mL)** | **IIEF-5 Score (Baseline)** | **IIEF-5 Score (Final)** | **Urinary Incontinence (% of men)** | **Need for transfusion (% of men)** | **Infection (% of men)** | **Urethral Stricture Rates (% of men)** | **Bladder Neck Contracture Rates (% of men)** | **Re-Operation Rate for Adenoma (% of men)** |
| --- | --- | --- | --- | --- | --- | --- | --- | --- | --- | --- | --- | --- | --- | --- | --- | --- | --- | --- | --- | --- | --- | --- | --- | --- | --- | --- |
| Tan, et. al. 2003 ^5^ | A randomized trial comparing holmium laser enucleation of the prostate with transurethral resection of the prostate for the treatment of bladder outlet obstruction secondary to benign prostatic hyperplasia in large glands (40 to 200 grams) | 12 months | EEP | 31 | Holmium laser | 25 | 71.7 | 77.8 | 0.74 ± 0.03 | 1.15 ± 0.11 | 26 ± 1.1 | 4.3 ± 0.7 | 4.8 ± 0.2 | 1.5 ± 0.5 | 8.4 ± 0.5 | 21.8 ± 2.1 | 113.5 |  |  |  | 0 | 0 |  | 3.33 |  |  |
|  |  |  | TURP | 30 | Monopolar | 27 | 70.3 | 70 | 1.87 ± 0.42 | 2.08 ± 0.23 | 23.7 ± 1.2 | 5 ± 0.9 | 4.7 ± 0.2 | 1.4 ± 0.3 | 8.3 ± 0.4 | 18.4 ± 2.8 | 126.7 |  |  |  | 3.8 | 3.3 |  | 10 |  |  |
| Kuntz et. al. 2004 ^6^ | Transurethral holmium laser enucleation of the prostate versus transurethral electrocautery resection of the prostate: a randomized prospective trial in 200 patients | 12 months | EEP | 100 | Holmium laser | 89 | 68.0 ± 7.3 | 53.5 ± 20.0 | 1.15 ± 0.4 | 2.2 ± 0.7 | 22.1 ± 3.8 | 1.7 ± 1.8 |  |  | 4.9 ± 3.8 | 27.9 ± 9.9 | 237 ± 163 | 5.3 ± 15.3 |  |  | 1.1 | 0 |  | 3.2 |  |  |
|  |  |  | TURP | 100 | Monopolar | 86 | 68.7 ± 8.2 | 49.9 ± 21.1 | 1.8 ± 0.9 | 3.6 ± 1.6 | 21.4 ± 5.2 | 3.9 ± 3.9 |  |  | 5.9 ± 3.9 | 27.7 ± 12.2 | 216 ± 177 | 26.6 ± 60.4 |  |  | 1.1 | 2 |  | 1.1 |  |  |
| Rigatti, et. al. 2006 ^7^ | Urodynamics after TURP and HoLEP in Urodynamically Obstructed Patients: Are there any differences at 1 year of follow-up? | 12 months | EEP | 52 | Holmium laser | 52 | 65.14 ± 7.3 | 60.3 ± 36.7 | 1.29 ± 0.54 | 2.46 ± 0.83 | 21.6 ± 6.7 | 4.1 ± 2.3 | 4.6 ± 1.1 | 1.4 ± 0.9 | 8.2 ± 3.2 | 25.1 ± 7.2 |  |  |  |  | 5.769 |  |  | 3.846 |  |  |
|  |  |  | TURP | 48 | Monopolar | 48 | 64.5 ± 6.4 | 56.2 ± 19.4 | 2.41 ± 0.73 | 3.58 ± 0.79 | 21.9 ± 7.2 | 3.9 ± 3.6 | 4.7 ± 1.0 | 0.8 ± 1.28 | 7.8 ± 3.6 | 24.7 ± 10.0 |  |  |  |  | 4.167 |  |  | 8.333 |  |  |
| Briganti, et. al. 2006 ^8^ | Impact on Sexual Function of Holmium Laser Enucleation Versus Transurethral Resection of the Prostate: Results of a Prospective, 2-Center, Randomised Trial. | 2 years | EEP | 60 | Holmium laser | 60 | 66.25 ± 6.9 | 73.3 ± 31.7 |  |  | 21.1 ± 6.9 | 3.6 | 4.4 ± 1.2 |  |  |  |  |  |  |  |  |  |  |  |  |  |
|  |  |  | TURP | 60 | Monopolar | 60 | 64.18 ± 7.2 | 58.2 ± 21.48 |  |  | 21.6 ± 7.1 | 3.7 | 4.5 ± 1.0 |  |  |  |  |  |  |  |  |  |  |  |  |  |
| Wilson et. al. 2006 ^9^ | A Randomised Trial Comparing Holmium Laser Enucleation Versus Transurethral Resection in the Treatment of Prostates Larger Than 40 Grams: Results at 2 Years | 2 years | EEP | 31 | Holmium laser | 22 | 71.7 ± 1.1 | 77.8 ± 5.6 |  |  | 26 ± 1.1 | 6.1 ± 1.0 | 4.8 ± 0.2 | 1.25 ± 0.2 | 8.4 ± 0.5 | 21 ± 2.0 |  |  |  |  |  |  |  | 3.23 |  |  |
|  |  |  | TURP | 30 | Monopolar | 26 | 70.3 ± 1.0 | 70 ± 5.0 |  |  | 23.7 ± 1.2 | 5.2 ± 0.8 | 4.7 ± 0.2 | 1.25 ± 0.2 | 8.3 ± 0.4 | 19.3 ± 2.2 |  |  |  |  |  |  |  | 10 |  |  |
| Gupta et. al. 2006 ^10^ | Comparison of standard transurethral resection, transurethral vapour resection and holmium laser enucleation of the prostate for managing benign prostatic hyperplasia of >40 g | 12 months | EEP | 50 | Holmium laser | 50 | 65.88 ± 10.1 | 57.9 ± 17.6 | 1.19 ± 0.85 |  | 23.4 ± 4.5 | 5.2 ± 0.17 |  |  | 5.15 ± 4.4 | 25.1 ± 1.06 | 112 ± 155.9 | <20 |  |  | 0 | 0 | 2 | 2 |  |  |
|  |  |  | TURP | 50 | Monopolar | 50 | 65.67 ± 7.5 | 59.8 ± 16.5 | 1.9 ± 0.71 |  | 23.3 ± 3.9 | 5.6 ± 0.32 |  |  | 4.5 ± 4.7 | 23.7 ± 1.58 | 84 ± 129.7 | <20 |  |  | 0 | 2 | 2 | 4 |  |  |
| Ahyai et. al. 2007 ^11^ | Holmium Laser Enucleation versus Transurethral Resection of the Prostate: 3-Year Follow-Up Results of a Randomized Clinical Trial | 3 years | EEP | 100 | Holmium | 75 | 68.0 ± 7.3 | 53.5 ± 20.0 |  |  | 22.1 ± 3.8 | 2.7 ± 3.2 |  |  | 4.9 ± 3.8 | 29 ± 11 | 237 ± 163 | 8.4 ± 16 |  |  |  |  |  | 4.1 | 3.1 | 1.3 |
|  |  |  | TURP | 100 | Monopolar | 69 | 68.7 ± 8.2 | 49.9 ± 21.1 |  |  | 21.4 ± 5.2 | 3.3 ± 3.0 |  |  | 5.9 ± 3.9 | 27.5 ± 9.9 | 216 ± 177 | 20.2 ± 33 |  |  |  |  |  | 3.3 | 3.3 | 0 |
| Montorsi et.al. 2008 ^12^ | Holmium Laser Enucleation Versus Transurethral Resection of the Prostate: Results From a 2-Center Prospective Randomized Trial in Patients With Obstructive Benign Prostatic Hyperplasia | 12 months | EEP | 52 | Holmium laser | 52 | 65.14 | 70.3 ± 36.7 | 1.29 ± 0.54 | 2.46 ± 0.83 | 21.6 ± 6.7 | 4.1 ± 2.3 | 4.6 ± 1.1 | 1.4 ± 0.9 | 8.2 ± 3.2 | 25.1 ± 7.2 |  |  |  |  | 1.7 | 0 |  | 1.7 |  |  |
|  |  |  | TURP | 48 | Monopolar | 48 | 64.5 | 56.2 ± 19.4 | 2.4 ± 0.73 | 3.58 ± 0.79 | 21.9 ± 7.2 | 3.9 ± 3.6 | 4.7 ± 1.0 | 0.8 ± 1.28 | 7.8 ± 3.6 | 24.7 ± 10 |  |  |  |  | 2.2 | 2.2 |  | 7.4 |  |  |
| Mavuduru et. al. 2009 ^13^ | Comparison of HoLEP and TURP in Terms of Efficacy in the Early Postoperative Period and Perioperative Morbidity | 9 months | EEP | 15 | Holmium laser | 14 | 69.86 ± 9.6 | 36.53 | 1.93 ± 0.59 |  | 22.53 ± 4.79 | 4.32 ± 1.25 |  |  | 5.79 ± 2.7 | 28.6 ± 6.2 | 91 ± 30 | 43 ± 10.61 |  |  | 7.1 | 0 |  | 0 |  |  |
|  |  |  | TURP | 15 | Monopolar | 13 | 66.46 ± 5.79 | 36.33 | 3.26 ± 0.74 |  | 21.4 ± 3.7 | 3.57 ± 1.03 |  |  | 6.9 ± 2.5 | 27.8 ± 6.5 | 103 ± 27 | 35.66 ± 15 |  |  | 0 | 6.7 |  | 6.7 |  |  |
| Eltabey et. al. 2010 ^14^ | Holmium laser enucleation versus transurethral resection of the prostate | 12 months | EEP | 40 | Holmium laser | 40 | 67.5 ± 8.1 | 62.4 ± 24.1 | 1.5 ± 1.4 | 2.6 ± 1.2 | 23 ± 3.6 | 2.2 ± 1.4 |  |  | 8.4 ± 2.3 | 24.9 ± 11.7 | 130 ± 96.5 | 5.3 ± 15.2 |  |  | 0 | 0 |  | 2.5 |  |  |
|  |  |  | TURP | 40 | Monopolar | 40 | 68.3 ± 9.2 | 58.5 ± 31.6 | 2.1 ± 1.1 | 3.8 ± 1.6 | 25 ± 5.1 | 3.7 ± 1.6 |  |  | 8.1 ± 2.7 | 25.5 ± 7.4 | 105 ± 89.7 | 24.1 ± 16.8 |  |  | 0 | 7.5 |  | 5 |  |  |
| Lusuardi et. al. 2011 ^15^ | Safety and Efficacy of Eraser Laser Enucleation of the Prostate: Preliminary Report | 6 months | EEP | 30 | Eraser laser | 30 | 66.5 ± 5.96 | 59.5 ± 15.13 | 1.37 ± 0.36 | 1.88 ± 0.62 | 26.9 ± 5.34 | 4.2 ± 1.06 | 5.1 ± 1.04 | 1.04 | 6.8 ± 2.43 | 21.57 ± 1.94 | 176.5 ± 75.39 | 35.27 ± 8.71 |  |  |  | 0 |  | 0 | 0 |  |
|  |  |  | TURP | 30 | Bipolar | 30 | 65.7 ± 6.2 | 59.1 ± 14.2 | 2.74 ± 0.57 | 3.8 ± 0.49 | 25.4 ± 4.8 | 4.43 ± 1.17 | 4.9 ± 1.00 | 1 | 6.4 ± 2.2 | 21.6 ± 1.57 | 180 ± 78.7 | 34.53 ± 8.85 |  |  |  | 0 |  | 0 | 0 |  |
| Fayad et. al. 2011 ^16^ | Holmium Laser Enucleation Versus Bipolar Resection of the Prostate: A Prospective Randomized Study. Which to Choose? | 6 months | EEP | 30 | Holmium laser | 30 | 60.67 ± 4.51 | 76.50 ± 17.22 |  | 2.04 ± 0.24 | 22.56 ± 2.45 | 5.5 ± 1.07 |  |  | 20.82 ± 1.21 |  |  | 20.3 ± 1.4 |  |  |  | 0 |  |  |  |  |
|  |  |  | TURP | 30 | Bipolar | 30 | 61.20 ± 4.21 | 80.60 ± 17.79 |  | 2 | 22.17 ± 2.35 | 5.33 ± 1.32 |  |  | 20.56 ± 0.92 |  |  | 25.6 ± 1.89 |  |  |  | 0 |  |  |  |  |
| Gilling et. al. 2012 ^17^ | Long-term results of a randomized trial comparing holmium laser enucleation of the prostate and transurethral resection of the prostate: results at 7 years | 7 years | EEP | 14 | Holmium laser | 14 | 71.70 ± 1.10 | 77.68 ± 32.13 |  |  | 26.39 ± 6.14 | 8.0 ± 5.2 | 4.79 ± 1.07 | 1.47 ± 1.3 | 8.28 ± 2.18 | 22.09 ± 15.47 |  | 20.3 ± 1.4 |  |  |  |  |  |  |  | 0 |
|  |  |  | TURP | 17 | Monopolar | 17 | 70.30 ± 1.00 | 70.00 ± 27.78 |  |  | 23.72 ± 6.44 | 10.3 ± 7.42 | 4.7 ± 1.1 | 1.31 ± 0.85 | 8.26 ± 2.18 | 17.83 ± 8.61 |  | 25.6 ± 1.89 |  |  |  |  |  |  |  | 17.6 |
| Swiniarski, et. al. 2012 ^18^ | Thulium laser enucleation of the prostate (TmLEP) vs. transurethral resection of the prostate (TURP): evaluation of early results | 1 month | EEP | 54 | Thulium:YAG laser | 54 | 68.3 ± 6.8 | 62.03 ± 23.7 | 2.1 ± 0.8 | 3.6 ± 0.9 | 20.38 ± 2.59 | 8.52 ± 4.99 | 4.7 ± 1.0 | 1.9 ± 1.3 | 7.73 ± 3.52 | 21.88 ± 9.62 | 166.2 ± 110.5 | 33.3 ± 35.1 |  |  | 1.85 | 0 | 3.7 | 5.56 | 0 |  |
|  |  |  | TURP | 52 | Bipolar | 52 | 69.3 ± 7.2 | 66.5 ± 22 | 2.0 ± 0.9 | 3.5 ± 0.8 | 20.85 ± 6.03 | 8.58 ± 4.06 | 4.9 ± 1.0 | 1.6 ± 0.9 | 8.57 ± 3.61 | 23.93 ± 7.78 | 152 ± 112.2 | 36.2 ± 28.2 |  |  | 7.69 | 3.85 | 7.69 | 0 | 0 |  |
| Zhu et. al. 2013 ^19^ | Electrosurgical Enucleation Versus Bipolar Transurethral Resection for Prostates Larger than 70 ml: A Prospective, Randomized Trial with 5-Year Followup | 5 years | EEP | 40 | Plasmakinetic | 31 | 64.1 ± 4.8 | 113.8 ±32.0 | 1.48 ± 0.24 | 3.0 ± 0.0 | 24.6 ± 3.4 | 3.32 ± 1.5 | 4.2 ± 1.0 | 1.0 ± 1.55 | 4.7 ± 3.0 | 26.45 ± 8.1 | 228.63 ± 136.49 | 4.36 ± 3.89 | 21 ± 3.8 | 23.13 ± 2.8 | 0 | 0 | 2.5 | 2.5 | 0 | 0 |
|  |  |  | TURP | 40 | Bipolar | 30 | 64.8 ± 3.9 | 109.4 ± 32.4 | 2.5 ± 0.24 | 4.0 ± 0.0 | 25 ± 3.4 | 4.9 ± 1.9 | 4.0 ± 0.9 | 1.8 ± 2.88 | 4.4 ± 3.1 | 22.07 ± 7.3 | 295.32 ± 157.63 | 14.39 ± 4.44 | 20.5 ± 4.1 | 23.37 ± 2.8 | 0 | 2.5 | 2.5 | 2.5 | 2.5 | 6.7 |
| Sun et. al. 2014 ^20^ | Holmium laser enucleation of the prostate versus transurethral resection of the prostate: a randomized clinical trial | 12 months | EEP | 82 | Holmium laser | 82 | 72.16 ± 7.53 | 55.11 ± 29.03 | 4.73 ± 2.11 | 11.37 ± 3.39 | 24.4 ± 3.78 | 4.95 ± 2.2 | 4.56 ± 0.67 | 1.57 ± 0.7 | 5.28 ± 1.88 | 19.77 ± 5.07 | 115.83 ± 102.57 | 12.66 ± 15.66 |  |  |  | 1.2 |  | 3.6 |  |  |
|  |  |  | TURP | 82 | Monopolar | 82 | 71.91 ± 7.53 | 56.22 ± 30.48 | 5.31 ± 3.16 | 11.82 ± 3.41 | 24.55 ± 3.86 | 7.48 ± 2.03 | 4.6 ± 0.66 | 1.84 ± 0.74 | 5.69 ± 1.42 | 18.18 ± 4.55 | 108.01 ± 115.83 | 23.22 ± 27.18 |  |  |  | 11 |  | 4.9 |  |  |
| Hamouda et. al. 2014 ^21^ | A comparative study between holmium laser enucleation of the prostate and transurethral resection of the prostate: 12-month follow-up | 12 months | EEP | 30 | Holmium laser | 30 | 68.3 ± 8.7 | 56.8 ± 14.4 | 1 ± 0 | 1.52 ± 0.83 | 22.3 ± 4.0 | 6.45 ± 2.54 |  |  | 5.9 ± 3.1 | 19.5 ± 3.1 | 160 ± 84.6 | 12.8 ± 10.9 |  |  | 3 | 0 | 6 | 0 |  |  |
|  |  |  | TURP | 30 | Monopolar | 30 | 65.6 ± 7.9 | 56.0 ± 18.4 | 2.47 ± 0.63 | 3.56 ± 0.79 | 22.1 ± 3.1 | 3.95 ± 2.11 |  |  | 6.9 ± 3.0 | 10.5 ± 1.9 | 212.2 ± 113.6 | 11.6 ± 9.0 |  |  | 0 | 6.67 | 13 | 0 |  |  |
| Fayad et. al. 2015 ^22^ | Holmium Laser Enucleation of the Prostate Versus Bipolar Resection of the Prostate: A Prospective Randomized Study. “Pros and Cons” | 12 months | EEP | 60 | Holmium laser | 51 | 60.85 | 68.15 |  |  | 23.2 ± 2.0 | 4.6 ± 1.0 |  |  | 7.0 ± 0.9 | 18.9 ± 0.6 |  |  |  |  | 0 | 0 |  | 0 |  |  |
|  |  |  | TURP | 60 | Bipolar | 55 | 60.35 | 67.2 |  |  | 23.4 ± 2.3 | 6.0 ± 1.8 |  |  | 6.6 ± 1.0 | 18.4 ± 1.4 |  |  |  |  | 0 | 3.3 |  | 6.7 |  |  |
| Geavlete et. al. 2015 ^23^ | Bipolar Vaporization, Resection, and Enucleation Versus Open Prostatectomy: Optimal Treatment Alternatives in Large Prostate Cases? | 12 months | EEP | 80 | Bipolar | 72 | 68.5 ± 8.50 | 122.6 ± 30.7 | 1.6 ± 0.9 | 2.5 ± 0.8 | 24.7 ± 3.3 | 4.2 ± 1.7 | 4.1 ± 1.1 | 0.9 ± 0.4 | 6.6 ± 1.6 | 25.6 ± 3.1 | 134.1 ± 86.8 | 19.7 ± 8.5 |  |  | 0 | 1.25 | 3.75 | 2.8 | 1.2 |  |
|  |  |  | TURP | 80 | Bipolar | 71 | 69.5 ± 7.2 | 121.8 ± 30.4 | 2.2 ± 1.3 | 3.2 ± 1.5 | 25.2 ± 3.2 | 4.4 ± 1.9 | 4.4 ± 1.3 | 0.9 ± 0.3 | 6.4 ± 2.2 | 24.9 ± 3.9 | 151.5 ± 103.1 | 27.6 ± 9.5 |  |  | 1.25 | 1.25 | 6.25 | 4 | 2.8 |  |
| Bozzini et. al. 2017 ^24^ | Thulium laser enucleation (ThuLEP) versus transurethral resection of the prostate in saline (TURis): A randomized prospective trial to compare intra and early postoperative outcomes | 3 months | EEP | 102 | Thulium:YAG laser | 72.5 ± 17.54 | 89.7 ± 45.1 |  | 1.3 ± 2.55 | 1.7 ± 2.73 | 19.7 ± 7.73 | 5.85 ± 4.23 |  |  |  |  | 120 ± 75.89 | 31.3 ± 8.55 |  |  | 0 | 0 |  | 1 |  |  |
|  |  |  | TURP | 106 | Bipolar | 70.7 ± 16.09 | 81.9 ± 39.4 |  | 4.8 ± 3.81 | 5.2 ± 3.98 | 18.6 ± 6.98 | 5.78 ± 5.29 |  |  |  |  | 112.9 ± 83.67 | 39.8 ± 9.81 |  |  | 0 | 2.8 |  | 3.8 |  |  |
| Jhanwar et. al. 2017 ^25^ | Outcomes of transurethral resection and holmium laser enucleation in more than 60g of prostate: A prospective randomised study. | 2 years | EEP | 72 | Holmium laser | 72 | 67.7 ± 7.4 | 75.6 ± 12.8 | 1.3 ± 0.2 | 1.7 ± 0.4 | 26.01 ± 2.08 | 5.01 ± 1.26 |  |  | 8.4 ± 1.48 | 26.1 ± 3.11 | 184.8 ± 32.2 | 17.22 ± 6.63 | 12.67 ± 1.73 | 12.72 ± 1.82 | 0 | 0 | 2.8 | 0 |  |  |
|  |  |  | TURP | 72 | Monopolar | 72 | 66.8 ± 7.8 | 74.5 ± 12.6 | 2.0 ± 0.6 | 2.3 ± 0.5 | 25.85 ± 2.22 | 5.00 ± 1.22 |  |  | 8.7 ± 1.02 | 24.9 ± 3.06 | 187.1 ± 38.5 | 19.22 ± 9.09 | 12.65 ± 2.36 | 12.52 ± 2.12 | 0 | 4.2 | 9.7 | 2.7 |  |  |
| Elshal et. al. 2020 ^26^ | Randomised trial of bipolar resection vs holmium laser enucleation vs Greenlight laser vapo-enucleation of the prostate for treatment of large benign prostate obstruction: 3-years outcomes | 3 years | EEP | 60 | Holmium | 54 | 66.2 ± 7 | 107 ± 21 |  |  | 19.7 | 0.8 |  |  | 7 | 29 |  |  | 21.9 | 21.9 |  | 0 |  |  |  | 5.6 |
|  |  |  | TURP | 62 | Bipolar | 55 | 66.1 ± 7 | 106 ± 23 |  |  | 14 | 1.7 |  |  | 9 | 22 |  |  | 20.0 | 20.5 |  | 6.5 |  |  |  | 30.9 |
| Shoji, et. al. 2020 ^27^ | Functional outcomes of transurethral thulium laser enucleation versus bipolar transurethral resection for benign prostatic hyperplasia over a period of 12 months: A prospective randomized study | 12 months | EEP | 70 | Thulium laser | 70 | 71.7 ± 5.5 | 58.9 ± 23.0 | 2.1 ± 0.6 | 2.4 ± 1.1 | 22.9 ± 5.1 |  | 5 ± 0.4 |  | 5.7 ± 2.1 |  | 112.1 ± 149.8 |  |  |  |  | 0 |  | 1.4 | 1.4 |  |
|  |  |  | TURP | 70 | Bipolar | 70 | 72.6 ± 6.5 | 56.1 ± 17.3 | 3.1 ± 0.8 | 3.2 ± 1.1 | 22.9 ± 5.1 |  | 5 ± 0.4 |  | 5.8 ± 2.4 |  | 116.0 ± 143.5 |  |  |  |  | 1.4 |  | 2.9 | 2.9 |  |
| El-Hawy, et. al. 2021 ^28^ | Two‑year follow‑up after holmium laser enucleation of the prostate and bipolar transurethral resection of the prostate: a prospective randomized study | 2 years | EEP | 59 | Holmium laser | 49 | 63.79 ± 5.6 | 74.5 ± 9.75 | 1.02 ± 0.06 | 1.4 ± 0.08 | 24.02 ± 3.56 | 5.87 ± 0.97 |  |  | 6.69 ± 2.4 | 24.9 ± 2.49 | 164.04 ± 31.2 | 27.55 ± 3.95 |  |  | 1.8 | 0 | 7.27 |  |  |  |
|  |  |  | TURP | 65 | Bipolar | 48 | 64.12 ± 6.9 | 75.02 ± 9.23 | 2.5 ± 0.21 | 1.5 ± 0.12 | 25.05 ± 4.1 | 5.83 ± 0.88 |  |  | 6.58 ± 2.1 | 24 ± 2.33 | 157.75 ± 32.4 | 29.43 ± 1.95 |  |  | 0 | 1.815 | 9.09 |  |  |  |
| Fuschi, et. al. 2022 ^29^ | B-TURP versus HoLEP: Peri-Operative Outcomes and Complications in Frail Elderly (>75 y.o.) Patients: A Prospective Randomized Study | 6 months | EEP | 96 | Holmium laser | 96 | 79.2 ± 0.92 | 76.13 ± 0.84 | 3.6 ± 0.23 | 2.9 ± 0.67 | 18 ± 0.23 | 8 ± 0.18 |  |  | 8.4 ± 0.12 | 18 ± 0.23 |  | 31.98 ± 12.05 |  |  |  | 0 |  | 2.1 | 2.1 |  |
|  |  |  | TURP | 104 | Bipolar | 104 | 80.1 ± 0.91 | 65.6 ± 0.89 | 6.9 ± 0.45 | 2.6 ± 0.55 | 19 ± 0.24 | 7 ± 0.19 |  |  | 8.6 ± 0.13 | 16.2 ± 0.22 |  | 40.96 ± 16.23 |  |  |  | 1 |  | 4.8 | 1 |  |
| Habib et. al. 2022 ^30^ | Holmium laser enucleation versus bipolar resection in the management of large-volume benign prostatic hyperplasia: A randomized controlled trial | 3 years | EEP | 57 | Holmium laser | 57 | 66.77 ± 6.78 | 128.66 ± 47.61 | 0.78 ± 0.21 | 0.92 ± 0.21 | 27.01 ± 4.98 | 4.57 ± 2.98 | 5 ± 1.52 | 1.65 ± 0.76 | 7.42 ± 3.14 | 29.23 ± 7.79 | 161.15 ± 119.85 | 27.09 ± 13.91 |  |  | 0 | 0 |  | 1.8 |  | 0 |
|  |  |  | TURP | 55 | Bipolar | 55 | 65.47 ± 7.98 | 119.12 ± 32.66 | 1.84 ± 0.92 | 1.76 ± 0.69 | 28.32 ± 3.78 | 7.60 ± 2.28 | 5 ± 1.52 | 2 ± 0 | 6.88 ± 2.19 | 21.05 ± 2.99 | 130.46 ± 70.83 | 36.02 ± 22.57 |  |  | 0 | 9.1 |  | 5.5 |  | 0 |
| Desai, et. al. 2024 ^31^ | A comparative evaluation of thulium laser enucleation versus bipolar transurethral resection of prostate in the management of large prostate (>60 g): A prospective randomized controlled trial | 3 months | EEP | 36 | Thulium laser | 36 | 67.69 ±5.81 | 79.78 ± 13.45 |  |  | 23.86 | 2.97 ± 0.95 |  |  | 8.35 | 18.88 ± 1.21 | 101.82 | 24.71 ± 6.76 |  |  |  | 0 |  | 0 | 0 |  |
|  |  |  | TURP | 36 | Bipolar | 36 | 68.06 ± 5.80 | 78.83 ± 15.98 |  |  | 23.52 | 4.61 ± 2.78 |  |  | 8 | 18.02 ± 2.79 | 116.04 | 29.89 ± 20.09 |  |  |  | 0 |  | 0 | 0 |  |
| Dhirubhai Tadha, et. al. 2024 ^32^ | A Prospective, Randomized Study Comparing the Outcome After Thulium Laser Enucleation of the Prostate with Conventional Monopolar TURP for the Treatment of Symptomatic Benign Prostatic Hyperplasia | 6 months | EEP | 35 | Thulium laser | 35 | 65.61 ± 8.56 | 69.74 ± 15.3 | 1.24 ± 0.27 | 1.79 ± 0.35 | 23.06 ± 4.22 | 2.17 ± 1.15 | 5.06 ± 0.68 | 0.34 ± 0.54 | 6.53 ± 1.63 | 28 ± 1.71 | 121.09 ± 63.45 | 9.8 ± 7.94 | 19.34 ± 4.7 | 19.45 ± 3.64 |  | 0 |  |  |  |  |
|  |  |  | TURP | 35 | Monopolar | 35 | 65.60 ± 8.25 | 70.09 ± 13.59 | 1.59 ± 0.35 | 2.1 ± 0.32 | 24.09 ± 2.36 | 2.23 ± 0.73 | 5.03 ± 0.71 | 0.29 ± 0.46 | 6.1 ± 1.63 | 26.8 ± 1.34 | 110.14 ± 62.82 | 8.1 ± 6.32 | 17.94 ± 3.23 | 18.23 ± 2.73 |  | 5.71 |  |  |  |  |

1. Tan AHH, Gilling PJ, Kennett KM, Frampton C, Westenberg AM & Fraundorfer MR. A randomized trial comparing holmium laser enucleation of the prostate with transurethral resection of the prostate for the treatment of bladder outlet obstruction secondary to benign prostatic hyperplasia in large glands (40 to 200 grams). Journal of Urology, 170(4 I), 1270-1274 (2003)
2. Kuntz RM, Ahyai S, Lehrich K, & Fayad A. Transurethral holmium laser enucleation of the prostate versus transurethral electrocautery resection of the prostate: A randomized prospective trial in 200 patients. Journal of Urology, 172(3), 1012-1016 (2004)
3. Rigatti L, Naspro R, Salonia A, Centemero A, Ghezzi M, Guazzoni G, et. al. Urodynamics after TURP and HoLEP in urodynamically obstructed patients: Are there any differences at 1 year of follow-up? Urology, 67(6), 1193-1198 (2006)
4. Briganti A, Naspro R, Gallina A, Salonia A, Vavassori I, Hurle R, et. al. Impact on Sexual Function of Holmium Laser Enucleation Versus Transurethral Resection of the Prostate: Results of a Prospective, 2-Center, Randomized Trial. Journal of Urology, 175(5), 1817-1821 (2006)
5. Wilson LC, Gilling PJ, Williams A, Kennett KM, Frampton CM, Westenberg AM, et. al. A Randomised Trial Comparing Holmium Laser Enucleation Versus Transurethral Resection in the Treatment of Prostates Larger Than 40 Grams: Results at 2 Years. European Urology, 50(3), 569-573 (2006)
6. Gupta N, Sivaramakrishna, Kumar R, Dogra PN, & Seth A. Comparison of standard transurethral resection, transurethral vapour resection and holmium laser enucleation of the prostate for managing benign prostatic hyperplasia of >40 g. BJU International, 97(1), 85-89 (2006)
7. Ahyai SA, Lehrich K, & Kuntz RM. Holmium Laser Enucleation versus Transurethral Resection of the Prostate: 3-Year Follow-Up Results of a Randomized Clinical Trial. European Urology, 52(5), 1456-1464 (2007)
8. Montorsi F, Naspro R, Salonia A, Suardi N, Briganti A, Zanoni M, et. al. Holmium Laser Enucleation Versus Transurethral Resection of the Prostate: Results From a 2-Center Prospective Randomized Trial in Patients With Obstructive Benign Prostatic Hyperplasia. Journal of Urology, 172(5 Pt 1):1926-1929 (2008)
9. Mavuduru RM, Mandal AK, Singh SK, Acharya N, Agarwal M, Garg S, et. al. Comparison of HoLEP and TURP in terms of efficacy in the early postoperative period and perioperative morbidity. Urologia Internationalis, 82(2), 130-135 (2009)
10. Eltabey MA, Sherif H, & Hussein AA. Holmium laser enucleation versus transurethral resection of the prostate. The Canadian journal of urology, 17(6), 5447-5452 (2010).
11. Lusuardi L, Myatt A, Sieberer M, Jeschke S, Zimmermann R, Hruby S, et. al. Safety and efficacy of eraser laser enucleation of the prostate (ELEP), Preliminary report. Journal of Endourology, 186(5), 1967-1971 (2011)
12. Fayad AS, El Sheikh MG, Zakaria T, Elfottoh HA, & Alsergany R. Holmium laser enucleation versus bipolar resection of the prostate: A prospective randomized study. which to choose? Journal of Endourology, 25(8), 1347-1352 (2011)
13. Gilling PJ, Wilson LC, King CJ, Westenberg AM, Frampton CM, & Fraundorfer MR. Long-term results of a randomized trial comparing holmium laser enucleation of the prostate and transurethral resection of the prostate: Results at 7 years. BJU International, 109(3), 408-411 (2012).
14. Świniarski PP, Stępień S, Dudzic W, Kęsy S, Blewniewski M, & Różański W. Thulium laser enucleation of the prostate (TmLEP) vs. transurethral resection of the prostate (TURP): evaluation of early results. Cent European J Urol, 65(3), 130-134 (2012)
15. Zhu L, Chen S, Yang S, Wu M, Ge R, Wu W, et. al. Electrosurgical enucleation versus bipolar transurethral resection for prostates larger than 70 ml: A prospective, randomized trial with 5-year followup. Journal of Urology, 189(4), 1427-1431 (2013)
16. Sun N, Fu Y, Tian T, Gao J, Wang Y, Wang S, et. al. Holmium laser enucleation of the prostate versus transurethral resection of the prostate: A randomized clinical trial. International Urology and Nephrology, 46(7), 1277-1282 (2014)
17. Hamouda A, Morsi G, Habib E, Hamouda H, Emam AB, & Etafy M. A comparative study between holmium laser enucleation of the prostate and transurethral resection of the prostate: 12-month follow-up. Journal of Clinical Urology, 7(2), 99-104 (2014)
18. Fayad AS, Elsheikh MG, Zakaria T, Elfottoh HA, Alsergany R, Elshenoufy A, et. al. Holmium Laser Enucleation of the Prostate Versus Bipolar Resection of the Prostate: A Prospective Randomized Study. "Pros and Cons". Urology, 86(5), 1037-1041 (2015)
19. Geavlete B, Bulai C, Ene C, Checherita I, & Geavlete P. Bipolar vaporization, resection, and enucleation versus open prostatectomy: Optimal treatment alternatives in large prostate cases? Journal of Endourology, 29(3), 323-331 (2015)
20. Bozzini G, Seveso M, Melegari S, de Francesco O, Buffi NM, Guazzoni G, et. al. Thulium laser enucleation (ThuLEP) versus transurethral resection of the prostate in saline (TURis): A randomized prospective trial to compare intra and early postoperative outcomes. Actas Urologicas Espanolas, 41(5), 309-315 (2017)
21. Jhanwar A, Sinha R, Bansal A, Prakash G, Singh K, & Singh V. Outcomes of transurethral resection and holmium laser enucleation in more than 60 g of prostate: A prospective randomized study. Urology Annals, 9(1), 45-50 (2017)
22. Elshal AM, Soltan M, El-Tabey NA, Laymon M, & Nabeeh A. Randomised trial of bipolar resection vs holmium laser enucleation vs Greenlight laser vapo-enucleation of the prostate for treatment of large benign prostate obstruction: 3-years outcomes. BJU International, 126(6), 731-738 (2020).
23. Shoji S, Hanada I, Otaki T, Ogawa T, Yamada K, Uchida T, et. al. Functional outcomes of transurethral thulium laser enucleation versus bipolar transurethral resection for benign prostatic hyperplasia over a period of 12 months: A prospective randomized study. International Journal of Urology, 27(11), 974-980 (2020)
24. El-Hawy MM, Eldakhakhny A, AbdEllatif A, Salem EA, Ragab A, Elsharkawy MS, et. al. Two-year follow-up after holmium laser enucleation of the prostate and bipolar transurethral resection of the prostate: a prospective randomized study. 27(1) (2021)
25. Fuschi A, Asimakopoulos AD, Scalzo S, Martoccia A, Al Salhi Y, Suraci PP, et. al. B-TURP versus HoLEP: Peri-Operative Outcomes and Complications in Frail Elderly (>75 y.o.) Patients: A Prospective Randomized Study. BIOMEDICINES, 10(12) (2022)
26. Habib E, Abdallah MF, ElSheemy MS, Badawy MH, Nour HH, Kamal AM, et. al. Holmium laser enucleation versus bipolar resection in the management of large-volume benign prostatic hyperplasia: A randomized controlled trial. International Journal of Urology, 29(2), 128-135 (2022)
27. Desai D, Gupta A, Goel H, Sharma U, Mehra K, & Katiyar V. A comparative evaluation of thulium laser enucleation versus bipolar transurethral resection of prostate in the management of large prostate (>60 g): A prospective randomized controlled trial. National Journal of Physiology, Pharmacy and Pharmacology, 14(2), 364-371 (2024)
28. Dhirubhai Tadha A, Sarkar D, & Kumar Pal D. A Prospective, Randomized Study Comparing the Outcome After Thulium Laser Enucleation of the Prostate with Conventional Monopolar TURP for the Treatment of Symptomatic Benign Prostatic Hyperplasia. Urol Res Pract, 50(1), 42-46 (2024)
